# Proteomics and phosphoproteomics to study Tuina reverses capsule fibrosis in frozen shoulder: a research report based on rats

**DOI:** 10.1038/s41598-023-50904-9

**Published:** 2024-01-05

**Authors:** Yingjie Qiao, Jianmin Wang, Lijun Zheng, Yanhong Yang, Huadong Li, Muzhen Li, Shidong Zhang, Hongyi Wang, Tiantian Zhang

**Affiliations:** 1 College of Acupuncture and Tuina, Shandong University of Traditional Chinese Medicine, Jinan, Shandong China; 2 Department of Tuina, Shandong University of Traditional Chinese Medicine Affiliated Hospital, Jinan, Shandong China; 3 Special Education and Rehabilitation College, Binzhou Medical University, Binzhou, Shandong China

**Keywords:** Proteomic analysis, Therapeutics

## Abstract

Frozen shoulder (FS) is a common disorder often treated with Tuina, but the mechanisms involved remain unclear. We employed proteomics and phosphoproteomics to investigate the mechanisms associated with the treatment of capsule fibrosis in FS rats. We used a method composed of three weeks of cast immobilization to establish a model of FS. We then administered Tuina once daily for 14 days, evaluated glenohumeral range of motion (ROM), assessed histological changes, and identified differentially expressed proteins (DEPs) using proteomics and phosphoproteomics. This study demonstrated that Tuina could improve glenohumeral ROM and reserve capsule fibrosis in FS rats. Proteomics revealed proteins regulated by Tuina belonging to the PI3K-AKT and ECM receptor interaction signaling pathways. Phosphoproteomics detected differentially phosphorylated proteins regulated by Tuina to be enriched in the MAPK signaling pathway. The combination of proteomics and phosphoproteomics for Protein–Protein Interaction (PPI) network analysis revealed that the phosphorylation of Myh3 and Srsf1 with a node degree larger than the average degree were considered the central regulatory protein modulated by Tuina to reverse capsule fibrosis. Thbs1, Vtn, and Tenascin-W were significantly enriched in PI3K-AKT and ECM receptor interaction signaling pathways and highly expressed in model rats. Tuina resulted in reduced expression of these proteins. Our findings demonstrated some of mechanisms behind the reversal of FS capsule fibrosis following Tuina, a scientific medical therapy for FS patients.

## Introduction

Frozen shoulder (FS), also known as adhesive capsulitis of the shoulder, is a multifaceted disease characterized by pain and dyskinesia. Epidemiological research demonstrated an average onset age of 50, ranging from 30 to 70 years old^1^. The prevalence of FS is approximately 2–5%, and the primary risk factors are diabetes, thyroid disease, stroke, and autoimmune diseases^2–4^. Studies have demonstrated that FS patients suffer from pain and reduced function, seriously impacting their quality of life, with symptoms such as insomnia and anxiety causing serious harm to their physical and mental health^5,6^.

FS is a debilitating disorder that can be cured in approximately 1–3 years following symptom onset, but occasionally symptoms do not completely subside^7^. Treatment is necessary and helpful for FS patients, with commonly used treatment approaches including corticosteroid injection, physical therapy, and arthroscopic surgery^8^. Complementary and alternative medicine (CAM) is commonly employed for the treatment of FS^9,10^. The traditional Chinese medicine (TCM) approach, Tuina, is a CAM method widely used in the clinical treatment of diseases that provides comfort, high patient compliance, safety, and no side effects^11^. Clinical research demonstrates that Tuina can effectively relieve pain, restore shoulder function, and enhance the quality of life of FS patients^12^. However, the associated mechanisms remain unclear. Our prior study confirmed that Tuina has excellent effects on FS patients^13^. Therefore, this study aims to reveal the some of mechanisms underlying the effects of Tuina.

Proteomics and phosphoproteomics can uncover protein activity in cells during disease progression, providing a theoretical basis for the elucidation of disease occurrence and progression, including early diagnosis and treatment^14,15^. In TCM, these technologies can be employed for the analysis of protein differences throughout various syndromes of the same disease to enrich current scientific knowledge, as well as reveal critical mechanisms for acupuncture and other TCM treatment strategies^16–18^. Our study represents an unprecedented examination of Tuina reversal of FS-associated capsule fibrosis via proteomic and phosphoproteomic analysis.

## Results

### The effectiveness of Tuina in improving glenohumeral range of motion and reversing capsule fibrosis

**Figure 1:**
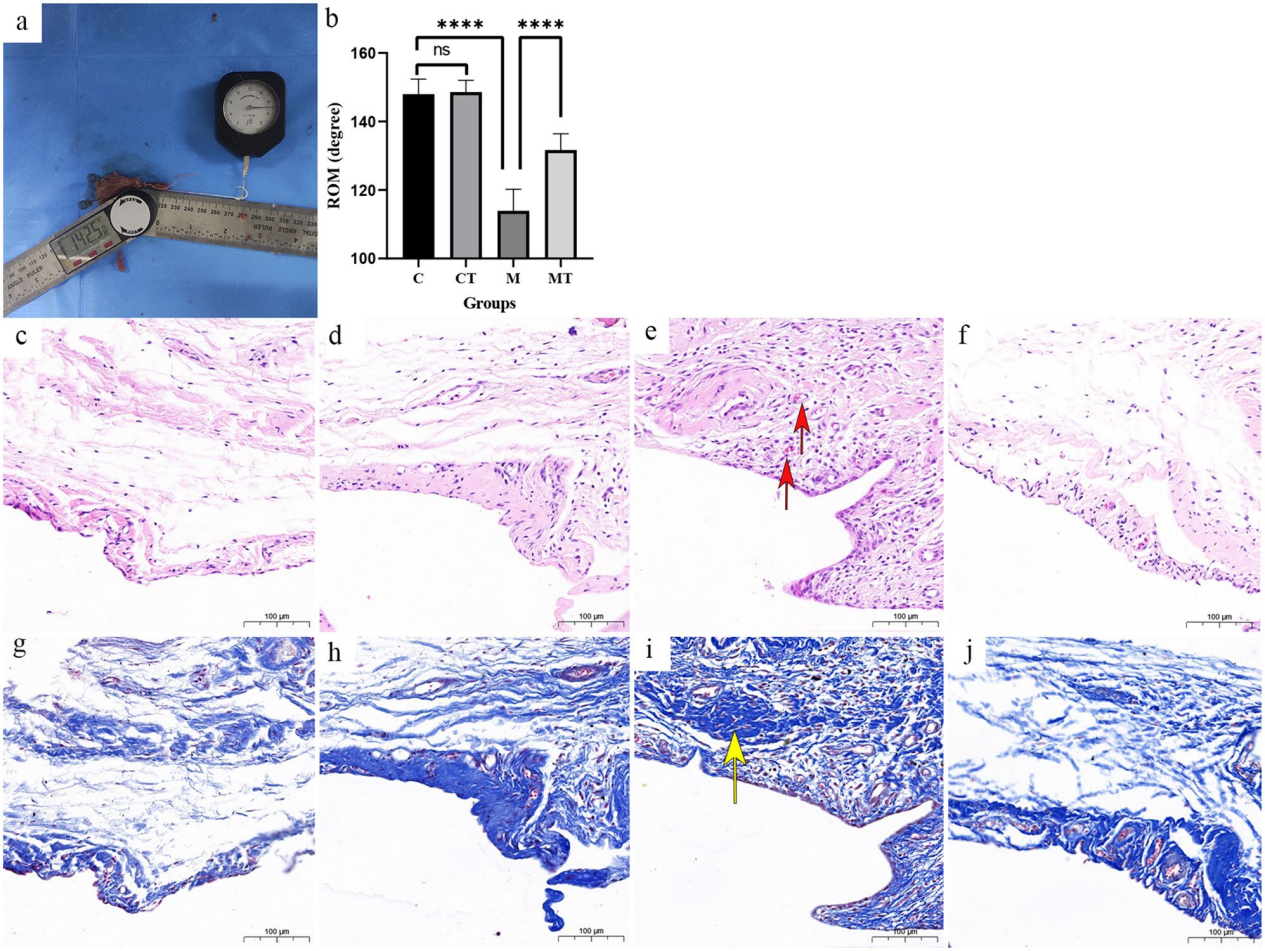
The efficacy of Tuina in FS rats. (**a**) Measurement of glenohumeral ROM. (**b**) Glenohumeral ROM across four groups of rats. Values are means ± S.D., n = 10. Significant differences are indicated by one-way ANOVA (ns P > 0.05,****p < 0.0001). (**c**,**g**) The normal capsule in C group (H&E and Masson staining). (**d**,**h**) The normal capsule in CT group (H&E and Masson staining). (**e**,**i**) The structure of the FS capsule in M group as follows: synovial hyperemia and capsule fibrosis (H&E and Masson staining). (**f**,**j**) The capsule is closely related to normal in MT group (H&E and Masson staining). Scale bar = 100 µm. Red arrow: erythrocyte stasis and vascular proliferation; Yellow arrow: fibrosis on histological findings.

The primary criterion for evaluating the effectiveness of Tuina in FS is the measurement of glenohumeral range of motion (ROM) (Fig. 1a). There were 4 groups in this study: the control group (C), the control combined with the Tuina treatment group (CT), the FS model group (M) and the FS model combined with the Tuina treatment group (MT). The details of these groups can be seen in Grouping Method secition. We observed that the average values of glenohumeral ROM were 147.9° ± 4.3° in the C group, 148.5° ± 3.4° in the CT group, 113.9° ± 6.3° in the M group, and 131.7° ± 4.7° in the MT group. As shown in the Fig. 1b, The difference between the glenohumeral ROM of rats in C group and CT group had no statistical significance (*P* > 0.05), and the ROM in the M group was significantly lower than that in the C group (*P* < 0.0001). Moreover, the ROM in the MT group and was significantly higher than that in the M group (*P* < 0.0001). The mitigating effect of Tuina on joint capsule fibrosis was observed through H&E staining and Masson staining. As illustrated in Fig. 1, H&E staining and Masson staining indicated the normal histological structure of the capsule in the C and CT groups (Fig. 1c, d, g, h). H&E staining revealed synoviocyte hyperplasia and synovial hyperemia in the M group, which are typical features of FS (Fig. 1e). These features were reduced to an extent after Tuina (Fig. 1f). Masson staining indicated the arrangement of fiber bundles throughout each group. The capsule was composed of a loose network of reticular fibers with fiber bundles organized in a neat direction. In the M group, the fiber bundles exhibited a disordered arrangement, suggesting capsule fibrosis (Fig. 1i). The capsules of rats in the MT group indicated that the fiber bundles were neatly and clearly stratified (Fig. 1j). These results were consistent with the results of our previous research^19^.

### Tuina reverses capsule fibrosis in frozen shoulder as indicated via protemocis

**Figure 2:**
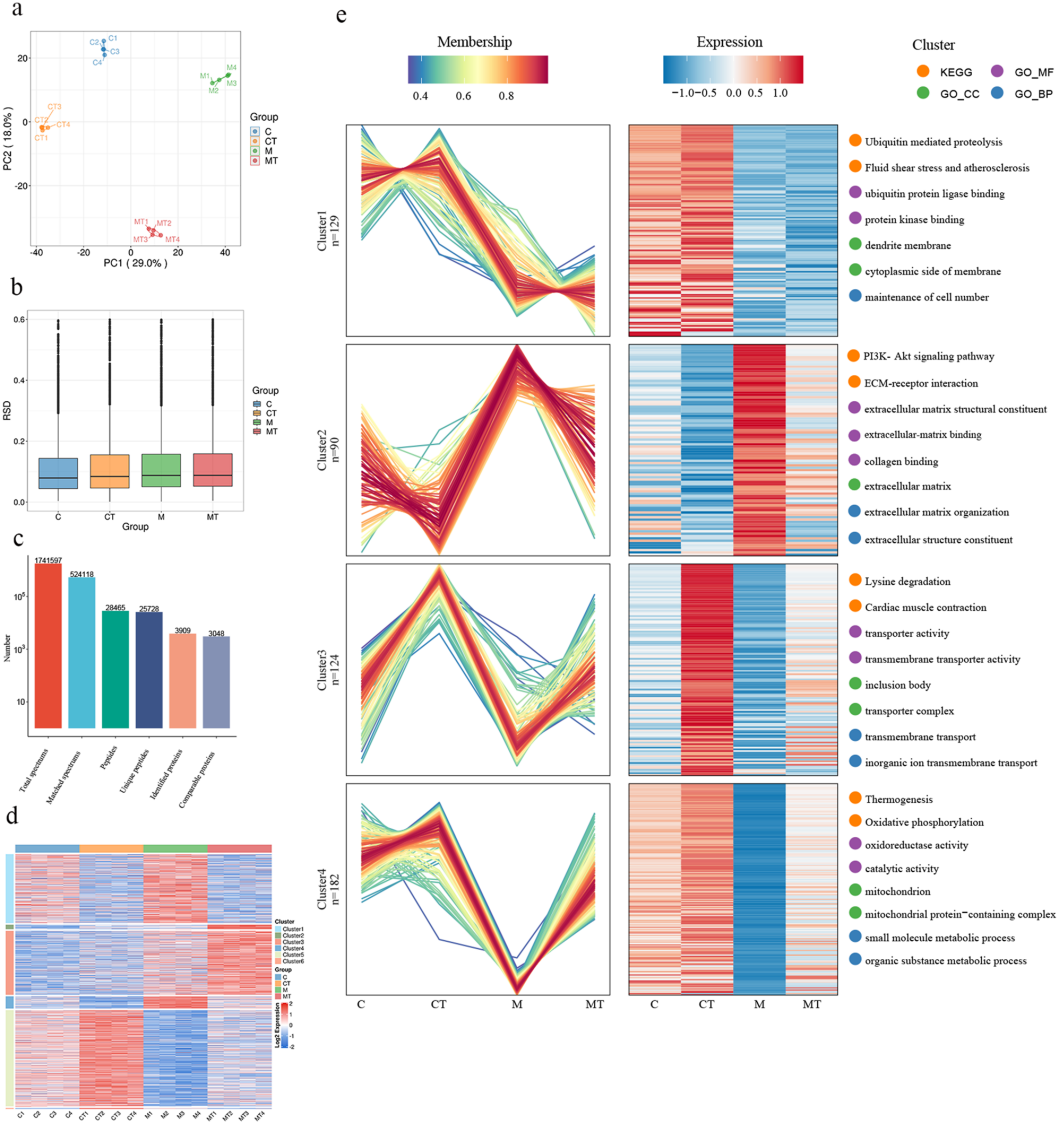
Proteomic findings and confirmation of key proteins. (**a**) PCA of proteomics. (**b**) RSD of proteomics. (**c**) Mass spectrometry fundamental statistics. (**d**) K-means clustering heatmap. (**e**) Mfuzz analysis of differential protein expression across the four groups. The shades red and blue represent high expression and low expression, respectively. Enrichment analysis is indicated on the right side of the illustrations.

Tuina can reverse fibrosis of the FS capsule, but its mechanisms has not been fully elucidated. In this study, some of mechanisms by which Tuina reverses fibrosis of the capsule was uncovered via proteomics. Before analysis, we tested the differences and repeatability of the samples in C, CT, M and MT groups. Principal component analysis (PCA) indicated that the degree of intragroup sample aggregation was high with limited differences, while the degree of sample aggregation between groups was low with pronounced differences (Fig. 2a). The assessment indicated that the value of relative standard deviation (RSD) is less than 0.2 which summarized samples in each group had adequate quantitative repeatability (Fig. 2b). As illustrated in Fig. 2c, we identified a total of 1,741,597 spectra based on 4D Label-free quantitative proteomics, comprising 524,118 matched spectra. A total of 28,465 peptides were identified, of which 25,728 represented unique peptides. In addition, we identified 3,909 proteins, of which 3,048 were comparable proteins (Table S1). Differential expression proteins (DEPs) analysis was conducted for k-means clustering (ANOVA *p*-value < 0.05), and the thermogram was drawn according to the clustering results (Table S2). This analysis characterized 1,820 DEPs separated into 6 clusters (Fig. 2d). The proteins selection for further analysis was based on the expression trends of DEPs: the proteins exhibiting the same expression trend in the CT/C and MT/M groups, but with an opposite expression trend in the M/C group were chosen. We thus selected 799 DEPs from Clusters 4 and 5 for Mfuzz expression clustering analysis.

As shown in Fig. 2e, following a Log2 ratio transformation, 525 DEPs with SD > 0.2 were chosen for Mfuzz expression clustering analysis (Table S3, S4, S5 and S6). In Cluster 2, the DEPs of group M were elevated compared to group C, and decreased following Tuina treatment. In Clusters 3 and 4, the DEPs of the M group decreased compared to the C group, and increased following Tuina administration. According to prior results, a large amount of type I (COL-I) and type III (COL-III) collagen deposition exists in the FS capsule tissue, mainly occurring in the extracellular matrix (ECM), which is reversed by Tuina acting on the ECM^20–22^. Therefore, we focused on DEPs associated with ECM regulation. In Cluster 2, the DEPs regulated by Tuina were enriched in the extracellular matrix, with GO terms denoting extracellular-matrix binding, extracellular-matrix structural constituent and collagen binding (molecular function), extracellular matrix organization, and extracellular structure organization (biological processes). Further analysis revealed that enriched KEGG pathways included the ECM-receptor interaction signaling pathway (Table S7). These analyses confirmed that Tuina was linked to changes in the ECM. Moreover, the PI3K-Akt signaling pathway showed the highest enrichment in the KEGG analysis.

### Tuina reverses capsule fibrosis in frozen shoulder as indicated via phosphoproteomics

**Figure 3:**
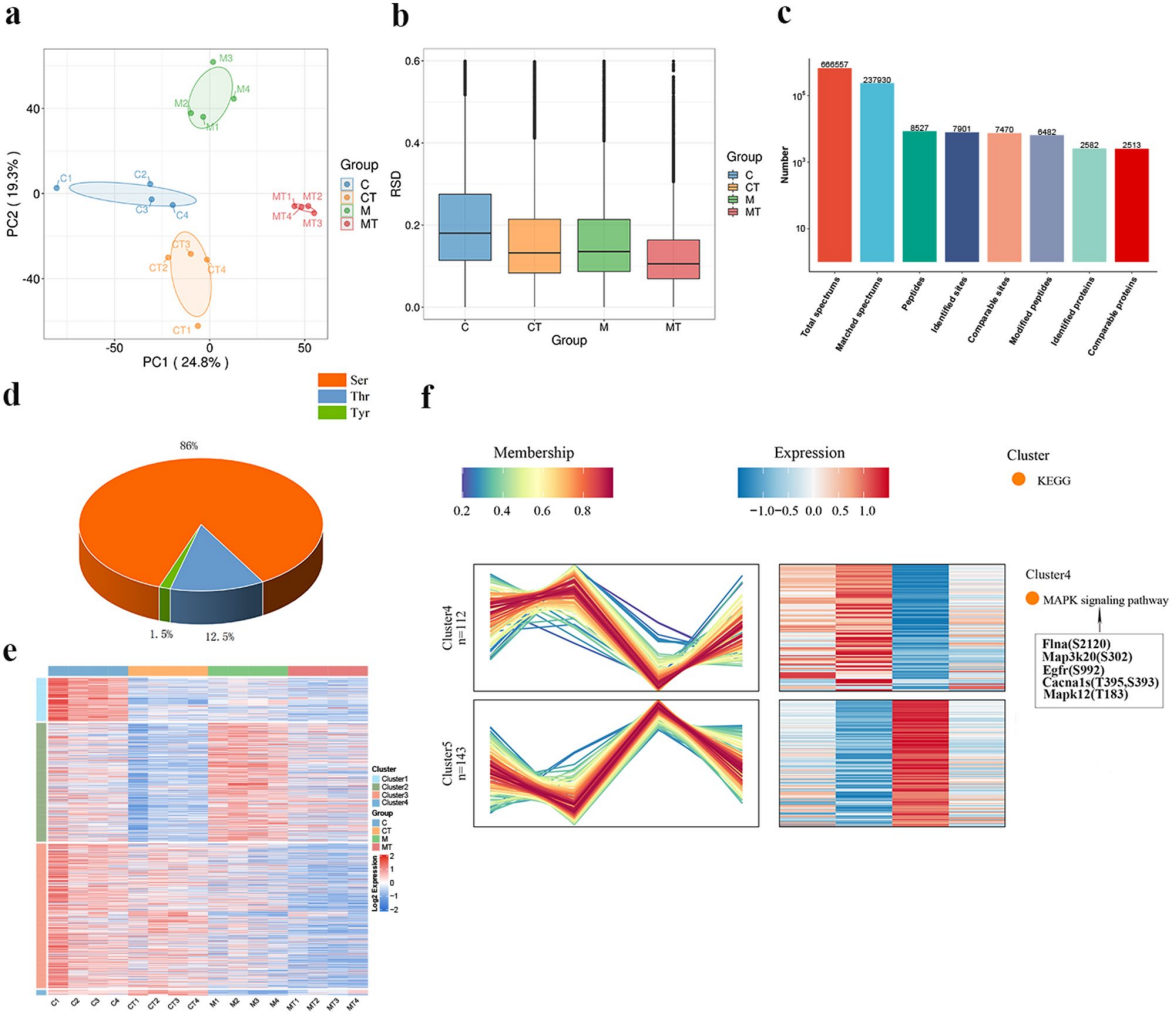
Phosphoproteomic analysis. (**a**) PCA of phosphoproteomics. (**b**) RSD of phosphoproteomics. (**c**) Mass spectrometry fundamental statistics. (**d**) Statistics of phosphorylation on serine, threonine, and tyrosine. (**e**) K-means clustering heatmap. (**f**) Mfuzz analysis of differential protein expression across the four groups. The shades of red and blue represent high expression and low expression, respectively. Enrichment analysis is depicted on the right side of the illustrations.

Protein phosphorylation is the most widespread protein modification process, and revealing the protein phosphorylation modification process in the reversal of FS capsule fibrosis via Tuina was accomplished through phosphoproteomics. PCA and RSD analysis indicated that the same results as proteomics, which means the samples in C, CT, M and MT groups showed good duplication within groups, significant differences between groups and adequate quantitative repeatability by phosphoproteomics (Fig. 3a, b). We generated a total of 2,582 proteins with 7,901 phosphorylation modification sites were identified through 4D Label-free quantitative phosphoproteomics (Fig. 3c). These numbers reflect the overall identification and detection depth of this project, and DEPs were screened based on comparable 7901 phosphorylation sites. In Fig. 3d (Table S8), of the 7,901 phosphorylation sites detected, 6,799 (86.0%) occurred on serine (pS), 984 (12.5%) occurred on Threonine (pT), and 118 (1.5%) occurred on tyrosine (pY). Differential protein expression analysis was conducted for k-means clustering (ANOVA p-value < 0.05), and the thermogram was constructed according to the clustering results (Table S9). The trend of expression within and between clusters following the enrichment of DEPs was unclear, and the protein differences within clusters were too large, making it unsuitable for preliminary protein screening (Fig. 3e). Therefore, all 7,901 differential phosphorylation sites were directly transformed via a Log2 logarithm, and the proteins with SD > 0.5 were screened.

Following screening, the remaining 916 phosphorylation sites were employed for clustering analysis of expression patterns using the Mfuzz method. Based on the trends of protein sites alterations, 7 clusters were grouped and performed the enrichment analysis (Table S10, S11, S12 and S14). As illustrated in Fig. 3f, in Cluster 4, the DEPs of group M increased relative to the C group, and decreased after Tuina treatment. In Cluster 5, the DEPs of the M group decreased in contrast with the C group, and increased following Tuina administration. We focused on the bioinformatics results in these two clusters. Representative results of KEGG enrichment analysis showed that Flna (S2120), Map3k20 (S302), Egfr (S992), Cacna1s (T395, S393) and Mapk12 (T183) were enriched in MAPK signaling pathway. MAPK signaling pathway also showed the highest enrichment in the analysis.

### Integration of proteomics and phosphoproteomics for protein–protein interaction networks analysis

Through Protein–Protein Interaction Networks (PPI), the proteomics and phosphoproteomics results were combined to assess and identify the central regulatory protein modulated by Tuina to reverse capsule fibrosis. For DEPs from phosphoproteomics and proteomics results, the difference threshold of significant upregulation was a differential expression change of more than 1.5, and the change threshold of significant downregulation was less than 1/1.5 to identify DEPs. Predicting differentially expressed protein interactions via STRING. By calculating the level of nodes and other parameters, we identified the key proteins playing an important role in Tuina reversing capsule fibrosis (Tables S15, S16, and S17). In this PPI network, the average node degree of DEPs in the CT/C group was 3.41. The average node degree of DEPs in the M/C group was 4.86. The average node degree of DEPs in the MT/M group was 4.52. And the DEPs with a node degree larger than the average degree were considered to have a substantial regulatory effect. Our study further analyzed proteins larger than the average node, and the findings indicated that there were a total of ten overlapping proteins and phosphorylation sites across the CT/C, M/C, and MT/M groups. In Table 1, Srsf1 (S199) and Csrp3 were upregulated in the M/C group, downregulated in the MT/M group, and downregulated in the CT/C group. Myh3 possessed four phosphorylation sites (S1916, S1148, S949, and T379) that were downregulated in the M/C group, upregulated in the MT/M group, and similarly upregulated in the CT/C group.

**Table 1:** Overlapping proteins in the CT/C, M/C, and MT/M comparison groups.

Gene encoding the protein	Amino acid	Site	CT/C		M/C		MT/M	
			Site type	Protein type	Site type	Protein type	Site type	Protein type
*Neb*	T	49	Down		Down		Down	
*Neb*	S	45	Down		Down		Down	
*Srsf1*	S	199	Down	Up	Up		Down	Up
*Myh3*	T	379	Up		Down	Up	Up	Down
*Myh3*	S	1916	Up		Down	Up	Up	Down
*Myh3*	S	1148	Up		Down	Up	Up	Down
*Myh3*	S	949	Up		Down	Up	Up	Down
*Myh7*	T	446	Up		Up		Down	
Unknown								
*Csrp3*				Down		Up		Down

### Validation of key proteins characterized via in proteomics research through qPCR and WB

Although multiple DEPs were found in proteomics and phosphoproteomics, we chosed to use DEPs based on previous literature. In prior RNA-seq-related studies, it has been determined that FS was closely related to the PI3K-AKT signaling pathway^23^. The findings of the KEGG pathway enrichment analysis of proteomics suggested a total of 9 DEPs enriched in PI3K-AKT signaling pathway. Of these, Thbs1, Vtn, and Tenascin-W were enriched in the ECM-receptor interaction signaling pathway and PI3K-AKT signaling pathway at the same time. Therefore, we selected these three proteins for further examination.

**Figure 4:**
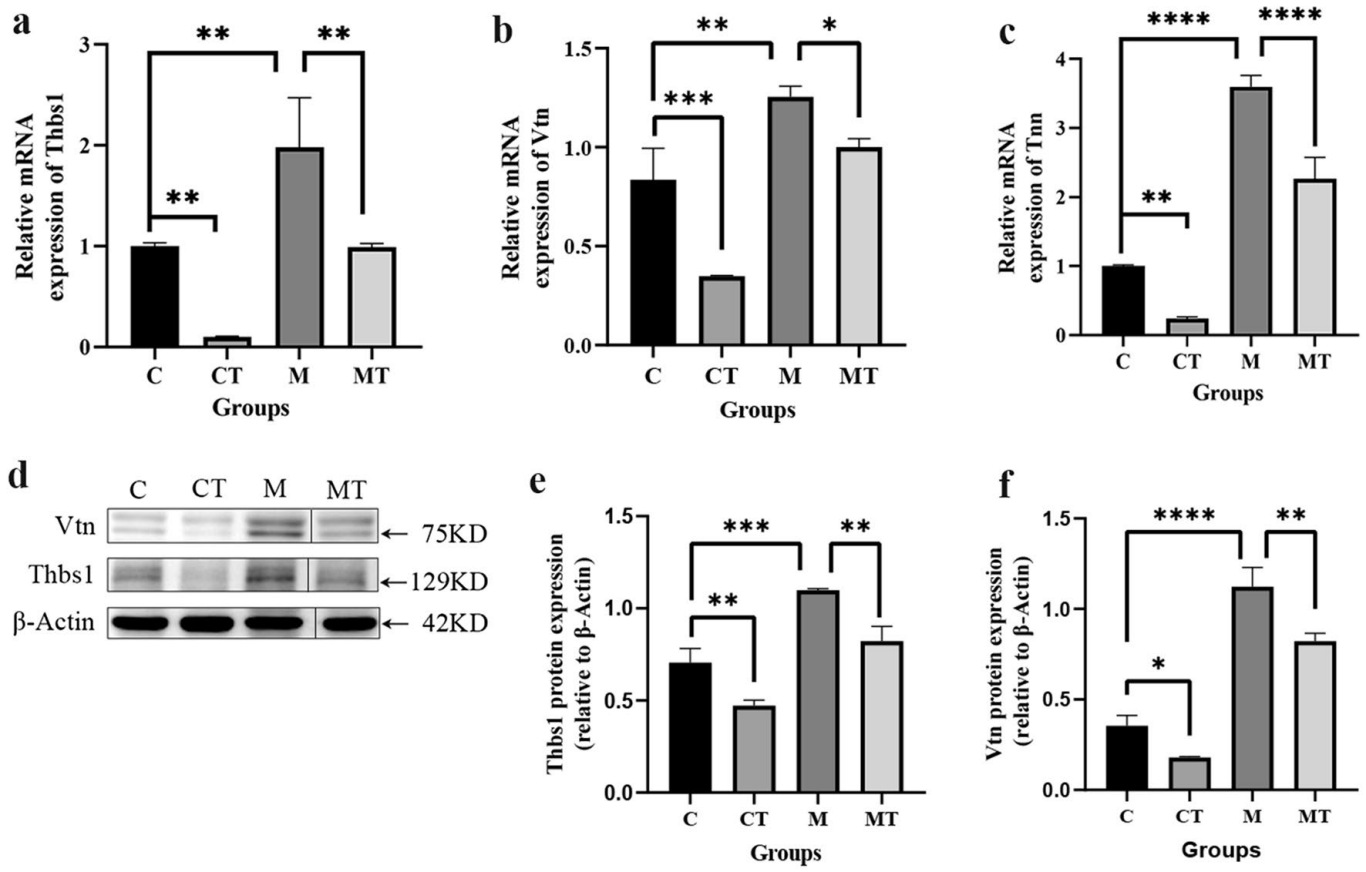
Validation of key proteins. (**a**–**c**) The expression of *Thbs1*, *Vtn*, and *Tnn* genes was quantified by qPCR. (**g**–**i**) Western blot analysis for Thbs1 and Vtn proteins. *p < 0.05, **p < 0.01, ***p < 0.001,****p < 0.0001. N = 3 rats/group. Original image of Western blot analysis was shown in the Supplementary Fig. 1.

In Fig. 4a–c, real-time quantitative polymerase chain reaction (qPCR) results demonstrated that the mRNA expression of *Thbs1*, *Vtn*, and *Tnn* in the M group was significantly up-regulated *(p* < 0.01, or *p* < 0.0001), and was inhibited following Tuina administration (*p* < 0.05, *p* < 0.01 or *p* < 0.0001). In the normal capsule, Tuina could also play the same regulatory role (*p* < 0.01, *p* < 0.0001). In Fig. 4d–f, further examination through Western Blot analysis (WB) demonstrated a significant upregulation of Thbs1 and Vtn proteins in the M group (*p* < 0.001, *p* < 0.0001) and inhibition following Tuina treatment (*p < *0.05, *p* < 0.01).

## Discussion

The most common clinical symptom of FS is the limited active and passive movement of the shoulder joint in all directions, which is difficult to overcome even with the use of pain relief drugs^24^. This illustrates that this limitation is predominantly caused by pathological changes in the shoulder joint, with pain being an important limiting factor of the resulting movement. Physiotherapy is commonly applied in the treatment of FS with satisfactory therapeutic results^25^. In traditional Chinese medicine, physiotherapy is often known as Tuina, which plays a crucial role in the treatment of FS. However, some studies have indicated that physical therapy cannot relieve persistent pain and suggested a steroid injection as primary therapy for FS^26^. Here, we investigated the underlying mechanisms of Tuina in reversing capsule fibrosis, providing a more reliable basis for future Tuina treatment. The treatments used in this study are based on our previous clinical and experimental researches^13,19^. In this study, we confirmed that Tuina can improve the shoulder joint function and reverse capsule fibrosis in the FS rat model.

In early FS stages, inflammation surrounding the capsule is the main contributing factor to shoulder pain, and with the continuous disease progress, the contracture and fibrosis of the capsule eventually result in limited activity of the shoulder joint and, ultimately, capsule fibrosis^27^. The capsule is composed of a thin inner synovial membrane and connective tissue with more fibers in the outer layer. Fibroblasts are the primary cell components in this connective tissue, producing ECM following tissue damage^28^. Pathological results indicated that Tunia reduces FS-related capsule fibrosis in rats, and we therefore focused on ECM regulation by Tuina in our study.

In contrast with traditional proteomics technology, 4D label-free quantitative proteomics is able to more quickly and efficiently identify and quantify various proteins, allowing an opportunity to investigate the mechanisms of Tuina therapy and improve clinical practice^29^. In this study, we analyzed the expression trends of DEPs across each group to assess the proteins directly regulated by Tuina. GO enrichment revealed that FS was closely associated with the biological process of ECM regulation (cluster 2) and that Tuina inhibits this process. KEGG pathway enrichment analysis showed that PI3K-AKT and ECM-receptor interaction were significantly enriched following Tuina in the FS rat model. We note this pathway is implicated in cell survival, growth, and apoptosis and regulates downstream targets, such as rapamycin (mTOR), hypoxia-inducible factor-1α (HIF-1α), and the FOX family^30^. Previous research indicated that IL-6 promotes AKT phosphorylation, activates the PI3-AKT signal pathway, increases the expression of COL-1 and COL-3, and results in the deposition of ECM and fibrosis in FS^31^. ECM-receptor interaction is a microenvironment pathway maintaining the structure and function of cells and tissues, which is located upstream of the PI3K-AKT pathway to act on it through various genes to generate different biological effects^32,33^. Moreover, genes linked to ECM-receptor interaction can result in collagen deposition in the ECM and produce fibrosis^34^. In this experiment, we identified that the genes *Vtn*, *Thbs1,* and *Tnn* were significantly enriched in the ECM-receptor interaction and the PI3K-AKT pathways and that the expression of these genes was increased in the FS rat model but could be limited by Tuina. We thus propose that Vtn, Thbs1, and Tenascin-W are target proteins in FS Tuina treatment.

Vtn, Thbs1, and Tenascin-W are important constituents of the extracellular matrix. Vtn is a glycoprotein in the extracellular matrix that is highly expressed in fibrotic tissue^35^. Vtn can mediate the regulation of collagens and elevate the expression of TGF-β to encourage fibrosis^36^. Similarly, Tenascin-W is a glycoprotein of the ECM, with four isoforms identified in mammals: Tenascin-C, -R, -W (-N), and -X. Tenascin-W is encoded by Tnn in rats, with a specific similarity to Tenascin-C being closely related to fibrosis, inflammation, and cancer^37^. Tenascin-W is also closely related to ECM production and tissue fibrosis^38^. Finally, Thbs1 is present in the ECM and can be secreted by various cell types, combining with ECM ligands to encourage TGF-β1 signaling in fibroblasts to form fibrotic tissue^39^. Thbs1 can be secreted by cyclic stretching force, bind with integrin receptors, transmit mechanical signals via YAP, and regulate the perception of the mechanical microenvironment of the ECM^40,41^. Vtn, Tenascins-w, and Thbs1 have not been previously reported in association with FS. Our qPCR findings confirmed that the mRNA levels of these genes were highly expressed in the FS model and suppressed by Tuina treatment in both normal and FS rat models. These observations were verified by Western Blot expression analysis of Vtn and Thbs1. However, due to a lack of antibodies, we did not analyze the expression of Tenascin-W. The specific functions of these proteins require further validation.

Phosphoproteomics was employed further to examine the mechanism of Tuina intervention on capsule fibrosis. The KEGG pathway enrichment analysis indicated that the DEPs modulated by Tuina had the highest level of enrichment in the MAPK pathway. The MAPK pathway is a classical pathway that includes four branching pathways: ERK, JNK, p38, and ERK5^42^. The study suggested that FS is closely linked to the abnormal mechanical force of the shoulder joint, and that mechanical force can increase the expression of MAPK molecules via integrin-β, with p44/42 MAPK (ERK) and JNK MAP being significantly increased in the capsule of FS patients^43^. p38 MAPK was not significantly expressed in the capsule, but P-p38 was significantly overexpressed in the hyperplastic synovium, suggesting that p38 participated in capsule fibrosis through phosphorylation^43^. Prior studies have indicated that Tuina can suppress inflammatory responses and alleviate pain by inhibiting the phosphorylation of p38^44^. This study determined that Tuina could promote the phosphorylation of diverse proteins and reverse capsule fibrosis through the MAPK pathway.

PPI network analysis indicated that the phosphorylation of Srsf1 and Myh3 played a role in the Tuina intervention of FS. This study found that in the FS model, the phosphorylation level of Srsf1 increased, and Tuina could reduce its phosphorylation, while the phosphorylation of Myh3 was inhibited at four sites, which Tuina could promote to intervene in frozen shoulders. Serine/arginine-rich splicing factor 1 (Srsf1) is a shear factor that primarily participates in mRNA splicing. Srsf1 belongs to the SR family of histones, and the in vivo activity of SR family proteins relies upon their phosphorylation state^45^. In fibroblasts, Srsf1 promotes the production, differentiation, and collagen deposition of EDA + Fn1 subtypes, playing a critical role in the formation of ECM, and is an important target for intervention in fibrosis-related diseases^46^. Myosin is a hexameric protein composed of a pair of myosin heavy chains and two pairs of light chains. It is a major contractile protein, which converts chemical energy into mechanical energy through ATP hydrolysis. Myosin heavy chain 3 (Myh3) is a member of the MYH family, which encodes a protein containing an IQ domain and myosin head domain^47^. It is highly expressed throughout embryonic and fetal development, and is an important part of myofilaments in skeletal muscle and nonmuscle cells^48^. Past studies have focused on the correlation between Myh3 and muscle-related disorders, but the role of Myh3 in FS has not been reported^49^. Our study found that Tuina could reverse capsule fibrosis by promoting Myh3 phosphorylation at S1916, S1148, S949, and T379, which indicated that Myh3 phosphorylation plays a crucial role in the mechanism of the treatment of Tuina therapy in FS. Therefore, the mechanism of Myh3 requires further study.

## Conclusion

Collectively, our results uncovered that Tuina could reverse capsule fibrosis. In addition, we elucidated some of mechanisms behind the reversal of FS following Tuina therapy, an effective complementary strategy for FS patients.

## Materials and methods

### Ethical policy

The study received approval from the Experimental Animal Ethics Committee of the Affiliated Hospital of Shandong University of Traditional Chinese Medicine (Number: AWE-2022-023). This study was conducted in accordance with relevant guidelines and regulations. All authors complied with the ARRIVE guidelines.

### Experimental animals

Pathogen-free male Sprague Dawley rats (aged seven weeks) weighing 250 g were procured from the Beijing Vital River Laboratory Animal Technology Co., Ltd under the animal use license number of SYXK (Lu) 20170022. All rats were raised in the Experimental Animal Center of the Affiliated Hospital of Shandong University of Traditional Chinese Medicine. The standard laboratory conditions consisted of alternating 12-h cycles of light and dark, a temperature of 20–24 °C, and 40–60% relative humidity. This study was performed in strict accordance with the recommendations in the Guide for the Care and Use of Laboratory Animals of the National Institutes of Health.

### Animal modeling

Following seven days of routine feeding, 42 rats were randomly separated into normal and model groups. The rats in the model group were anesthetized via intraperitoneal injection of tribromoethanol (250 mg/kg). Thereafter, the entire right arm, including the shoulder and chest, was immobilized using a plaster bandage^50^, which was removed after the right shoulder had been fully adducted and internally rotated for a period of 21 days. The procedure included proper immobilization, but the rats could self-feed and locomote normally. After modeling, the physical activity of rats was examined to assess the FS model. The stiffness of the right shoulder joint, and contracture signals in the right forelimb, accompanied by muscle atrophy, and an unsteady gait, were observed in the model organism. These characteristics confirm the successful induction of FS. In this study, the modeling success rate was 100%, achieved through the observation of physical activities. Each group contributed one rat for histopathological observation to further confirm the success of the FS model ().

### Grouping method

After evaluation, the rats of the normal group were randomly separated into the C group (n = 10) and the M group (n = 10). The model group rats were randomly separated into the M group (n = 10) and the MT group (n = 10). From days 22 through 35, we administered Tuina to the animals of groups CT and MT once daily. The groups of rats, including the C group and M group, were held and fixed, with the time matching the duration of the Tuina operation.

### Tuina treatment method

Tuina was performed according to our previous research^19^. Before administering Tuina, the operator held the rat in his left hand for 2 min. This ensured the animals were calm prior to the manipulation. Tuina administration was as follows: Firstly, the operator kneaded the right shoulder, scapula, and humerus muscles of the rat with his right thumb for 3 min. The operator then point-pressed the four acupoints of Jianyu, Jiquan, Tianzong, and Quchi with the vertical point of the thumb 30 times per acupoint. Finally, the right scapula of the rat was fixed with the left thumb, the forelimb was held with the right hand, and the shoulder joint was stretched for 10 s in the adduction, abduction, anterior extension, and posterior extension positions.

### Measurement of glenohumeral ROM

After the animals were sacrificed, the right scapula and proximal two-thirds of the humerus were removed. A thin thread is attached to an injection needle inserted into the humeral shaft and pulled at the other end with a 5 g force to make it parallel to the humeral shaft. The angle between the lower edge of the scapula and the humeral shaft is measured as glenohumeral ROM.

### Sample extraction and preservation

After measurement, a total of three samples from each group were fixed in 4% paraformaldehyde for three days, followed by decalcification in an EDTA solution (pH 7.2) for two months. The capsules of the remaining samples were dissected from the joint and stored at − 80 °C.

### Histology evaluation

Specimens were embedded in paraffin and cut into 5 μM thick slices. Standardized 5-μm thickness sections were stained using hematoxylin and eosin (H&E) and Masson solutions. The H&E staining methods included dewaxing and hydration, hematoxylin staining, eosin re-staining, dehydration, and clearing, followed by sealing. Masson staining includes dewaxing and hydration; staining with Bouin, azure blue, Mayer hematoxylin Fuchsin, Phosphomolybdate Acid, and Aniline Blue; dehydration and clearing, followed by sealing. Images were collected using a fluorescence inverted microscope (Leica, Leica DMIL LED).

### 4D label-free proteomic analysis

A total of 16 capsule samples (4 per group) were ground with liquid nitrogen into cell powder and transferred to a 5-mL centrifuge tube. After this, four volumes of lysis buffer (1% SDS, 1% protease inhibitor cocktail, and 1% phosphorylase inhibitor) were added to the cell powder, followed by sonication (220W, with a pulse of 3 s on and 5 s off, 3 min) on ice using a high-intensity ultrasonic processor (Scientz). The remaining debris was eliminated through centrifugation at 12,000 × *g* at 4 °C for 10 min. Finally, the supernatant was collected, and the protein concentration was determined using the BCA kit (Beyotime) following the manufacturer’s instructions. An aliquot of 20 μL of sample per lane was separated through SDS-PAGE and stained with Coomassie Blue.

An equal volume of each protein sample was adjusted to be consistent with the lysate. One volume of 4 °C precooled acetone was included with the protein solution, as well as 4 volumes of precooled acetone following vortex mixing. The mixture was precipitated at − 20 °C for 2 h. The precipitate was eliminated by centrifugation at 4500 × g for 5 min and washed twice with precooled acetone. After drying, 200 mM Tetraethylammonium bromide (TEAB) was added to the precipitate and dispersed using a high-intensity ultrasonic processor. A 1:50 trypsin-to-protein mass ratio was then used for digestion overnight. After digestion, the protein solution was reduced using 5 mM dithiothreitol for 30 min at 56 °C and alkylated using 11 mM iodoacetamide for 15 min at room temperature and maintained in the dark.

After being dissolved in solvent A (0.1% formic acid, 2% acetonitrile in water), the tryptic peptides were separated using the EASY-nLC 1200 Ultra Performance Liquid Chromatography system (Thermo Fisher Scientific). The flow rate was maintained at 500 nL/minute, where the mobile phase B consisted of a solution containing 0.1% formic acid and 90% acetonitrile in water. The gradient settings were: 0–68 min, 6% to 23% B; 68.0–82.0 min, 23% to 32% B; 82.0–86.0 min, 32% to 80% B; 86.0–90.0 min 80% B. The peptides were separated by an ultra-high performance liquid phase system and injected into an NSI ion source for ionization followed by mass spectrometry using an Orbitrap Exploris™ 480 (Thermo Fisher Scientific). The ion source voltage was set to 2.3 kV, and the peptide parent ions and their secondary fragments were detected and analyzed using a high-resolution Orbitrap. The primary mass spectrometry scan range was set to 400 to 1200 m/z, the scan resolution was set at 60,000, while the scanning range of secondary mass spectrometry was fixed at 110 m/z, the resolution of secondary scanning was set to 15,000, and TurboTMT was turned Off. The data acquisition mode adopted a data-dependent scanning (DDA) program (the top 25 peptide parent ions sequentially entered into the HCD collision pool for fragmentation using 27% of the fragmentation energy). The secondary mass spectrum data was retrieved by the Proteome Discoverer (v2.4.1.15).

The raw LC–MS datasets were first searched against database and converted into matrices containing LFQ intensity (the raw intensity after correcting the sample/batch effect) of proteins. The LFQ intensity (I) was transformed to the relative quantitative value (R) after centralization. The formula was listed as follow where i represented sample and j represented protein:

1 $$R_{ij} = \, I_{ij} / \, Mean\left( {I_{j} } \right)$$

We employed one-way analysis of variance (ANOVA) to screen all differentially expressed proteins (DEPs) with *p* < 0.05 for K-means clustering. The DEP expression trends were used to divide the differential proteins into multiple clusters and selection for further analysis. Following Log2 ratio transformation, DEPs with SD > 0.2 were selected for Mfuzz clustering analysis. The clustering results were used for gene ontology (GO) and Kyoto Encyclopedia of Genes and Genomes (KEGG) enrichment analyses. Significant enrichment was established if *p* < 0.05 using a Fisher's test. The process of GO annotation involves using the eggnog-mapper software (v2.1.6) to extract GO IDs from the identified proteins based on the EggNOG database, and then performing functional classification annotation analysis on the proteins according to cellular components, molecular functions, and biological processes^51^..Protein pathways based on the KEGG pathway database, and identify proteins through BLAST comparison (blastp, evalue ≤ 1e−4), for each sequence, the annotation is based on the top-scoring comparison result^52,53^.

### 4D label-free phosphoproteomics analysis

The protein extraction and trypsin digestion process were the same as those in proteomics. Peptide mixtures were incubated with an IMAC material with vibration in a loading buffer (50% acetonitrile and 0.5% acetic acid). To remove non-specifically adsorbed peptides, the IMAC materia were rinsed with 50% acetonitrile/0.5% acetic acid and 30% acetonitrile/0.1% trifluoroacetic acid solutions, sequentially. An elution buffer containing 10% NH_4_OH was added to elute the enriched phosphopeptides, and the enriched phosphopeptides were eluted through vibration. The supernatant containing phosphopeptides was obtained and lyophilized for LC–MS/MS analysis. The same process and parameter settings as in proteomics were employed in LC–MS/MS analysis, except the following gradient settings were used: 0–70 min, 3% to 18% B, 70.0–82.0 min, 18% to 28% B; 82.0–86.0 min, 28% to 80% B; 86.0–90.0 min, 80% B. The peptide segments are separated by an ultra-high performance liquid phase system and injected into the NSI ion source for ionization, before entering Orbitrap Exploris™ 480 (Thermo Fisher Scientific) was used for analysis. The ion source voltage is set to 2.2 kV, the FAIMS compensation voltage (CV) is set to −65 V, −45 V, and the peptide parent ions and their secondary fragments are detected and analyzed using high-resolution Orbitrap. The resolution of the secondary scanning was 30,000.

The raw LC–MS datasets were first searched against database and converted into matrices containing intensity of peptides across samples. The relative quantitative value of each modified peptide was then calculated based on these intensity information by the following steps: Firstly, the intensities of modified peptides (I) were centralized and transformed into relative quantitative values (R). Formula wa listed as follow where i denoted the sample and j denoted the modified peptid:

2 $$Rij = \, Iij/ \, Mean(Ij)$$

If both Proteomics and Post-translational modification profiling were conducted on the same cohort, the relative quantitative value of the modified peptide was usually divided by the relative quantitative value of corresponding protein to remove the influence from protein expression of modifications.Then identical bioinformatic analysis was employed as in proteomics for phosphoproteomics analysis.

### Integration of proteomics and phosphoproteomics for PPI analysis

The quantitative analysis results of proteomics and phosphoproteomics was analyed by PPI analysis. Three or more repetitions: Firstly, the samples to be compared were selected in pairwise, and the fold change (FC) was calculated as the ratio of the mean intensity for each protein or modification site in two sample groups. For example, to calculate the fold change between sample A and sample B, the formula was listed as following wher R denoted the relative quantitative value of the proteins or modification sites, i denoted the sample and k denoted the proteins or modification sites:

3 $$FC_{A/B,k} = \, Mean(Rik, \, i \in A) \, / \, Mean(Rik, \, i \in B)$$

To calculate the significance of the difference between groups, student's T test was performed on the relative quantitative value of each protein or modification site in the two sample groups. P value < 0.05 was considered as significant. The relative quantitative value of proteins or modification sites was log2 transformed. The formula is listed as following:

4 $$Pik = \, T.test(Log2(R_{ik} , \, i \in A), \, Log2(R_{ik} , \, i \in B)).$$

The proteins or modification sites with P value < 0.05, the fold change > 1.5 was regarded as significantly up-regulated protein, while P value < 0.05, the fold change < 1/1.5 was regarded as significantly down-regulated protein. After comparisons to the STRING protein network interaction database, proteins or modification sites with a fold change (FC) > 1.5 (or < 0.6667) and p < 0.05 for interaction relationships with confidence score > 0.7 (high confidence) were extracted.

### RNA extraction and real-time quantitative polymerase chain reaction

Total RNA was extracted from rat shoulder capsules using SparkZol. The reverse transcription reactions were performed using a SPARKscript II RT Plus Kit. Real-time PCR was conducted using a 2 × SYBR Green qPCR Mix. The database was acquired using a LightCycler 480 real-time PCR instrument (Roche). The primer sequences are denoted in Table 2.

**Table 2:** The primer sequences used for qPCR.

Gene name	Primer name	Bidirectional primer sequence 5′–3′	Product length /bp
*GAPDH*	GAPDH-F	CATGACCACAGTCCATGCCA	104
	GAPDH-R	CAGGGATGATGTTCTGGGCT	
*Tnn*	Tnn-F	AAGCGTTGGCGGAGTTATGTAGAAG	148
	Tnn-R	CGTAGGCGGATTCATTGGCAGTC	
*Thbs1*	Thbs1-F	CCGCCGATTCCAGATGATTCCTC	120
	Thbs1-R	GCAAGTCCAGGGTCACAGTTTACAG	
*Vtn*	Vtn-F	CAGCAGGGATTGGCATGGTGTAC	148
	Vtn-R	TCCTCGGCGTGAACGGTAGC	

### Western blot analysis

Proteins were pulverized using liquid nitrogen. After lysate centrifugation, the supernatant was examined using a bicinchoninic acid (BCA) assay kit (Thermo Fisher Scientific, Rockford, IL, USA). The protein samples were separated through electrophoresis and transferred to a PVDF membrane. The membranes were incubated with primary antibodies targeting Thbs1 (Bioss, bs-2715R, 1:500), Vtn (proteintech, 15833-1-AP, 1:6000), or β-Actin (bioss, bs-0061R, 1:2000), and diluted in a blocking solution overnight at 4 °C. This was followed by incubation with Goat Anti-Rabbit IgG (H + L) HRP (Sparkjade, EF0002, 1:5000). The protein bands were assessed using a Tanon 5200 imaging analysis system (Tanon Science & Technology Co. Ltd., Shanghai, China). Density analysis of relative protein levels was conducted using Image J software.

### Statistical analysis

SPSS 26.0 was utilized for statistical analysis. Differences between groups were analyzed by one-way analysis of variance (ANOVA). A value of *p* < 0.05 was considered to be statistically significant.

### Supplementary Information

Supplementary Tables.

Supplementary Figures.

## Data Availability

The mass spectrometry proteomics data have been deposited to the ProteomeXchange Consortium via the PRIDE partner repository with the dataset identifier PXD047467 and PXD047468. And all datasets generated during the current study are available from the corresponding author on reasonable request.
