# Supplementary material for: Proteomics and phosphoproteomics to study Tuina reverses capsule fibrosis in frozen shoulder: a research report based on rats
Source: Sci Rep. 2024 Jan 5;14:661. doi: 10.1038/s41598-023-50904-9 (PMC10770356; doi:10.1038/s41598-023-50904-9)
Supplement: Supplementary file 2 — Supplementary Figures. [file 41598_2023_50904_MOESM2_ESM.pdf]

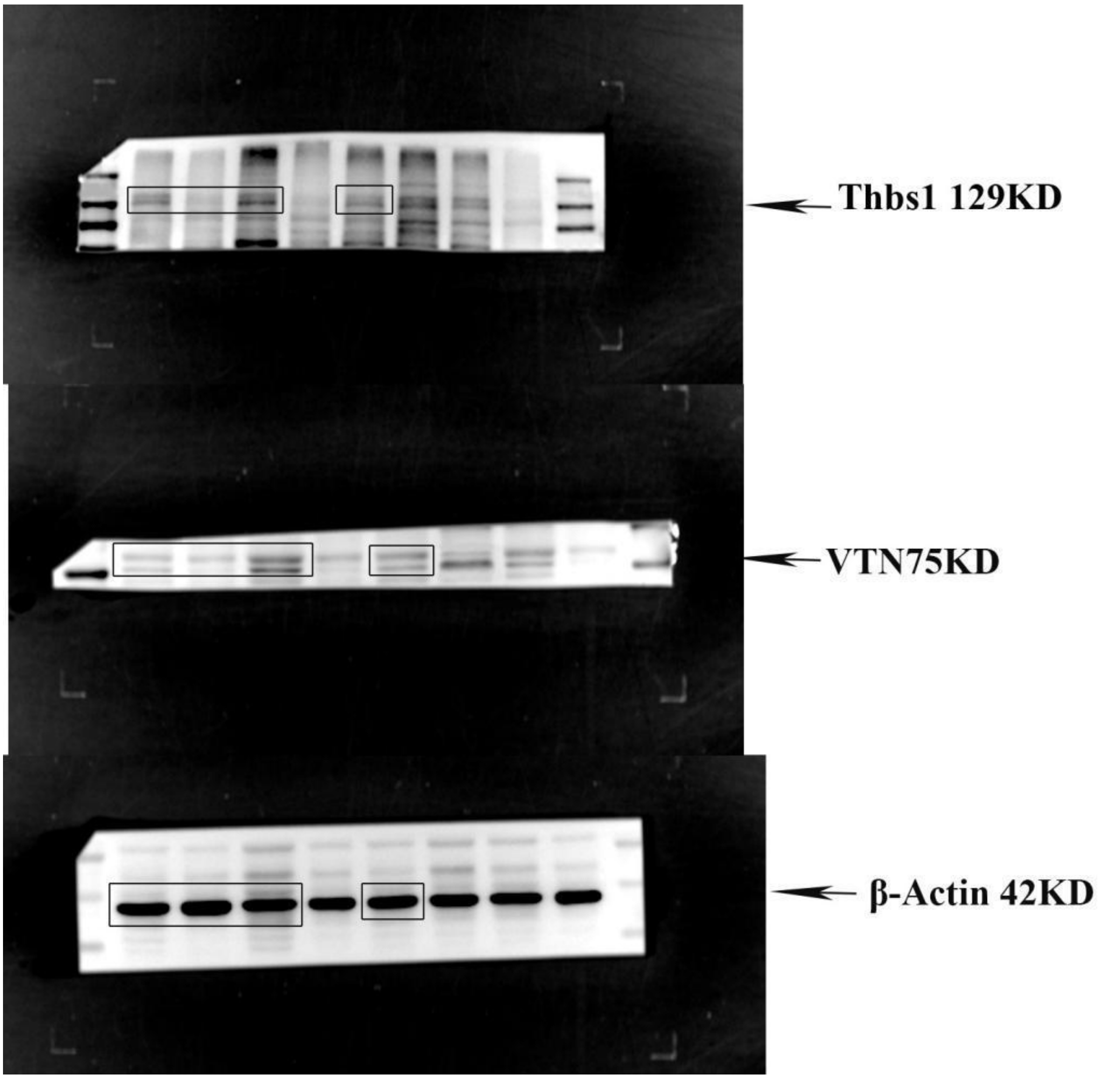

Supplementary figure 1. Blots of the cropped image shown in Figure 4g. To reflect differences, the order of loading samples is as follows: C, CT, M,CT, MT, M, MT, C. The cropped areas are labelled with balk-boxes(C, CT, M, MT). Each blots were cropped from the same gel.

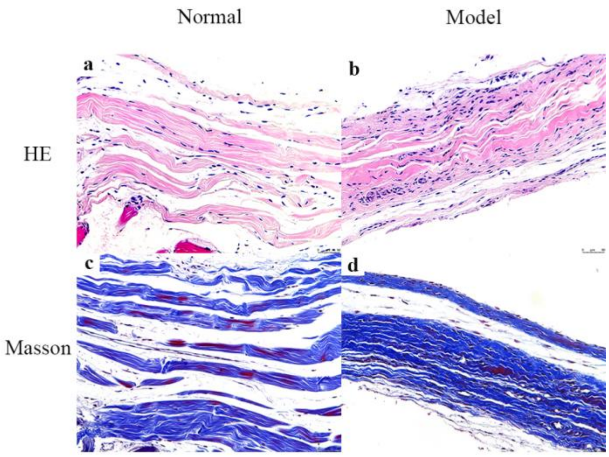

Supplementary figure 2. Pathological changes in the capsule of normal and model rats. (a,b) H&E staining (Scale bar: 50 μm). (c,d) Masson staining (Scale bar: 50 μm). H&E staining and Masson staining of the capsule demonstrated that the normal capsule was made up of a loose network of reticular fibers in normal rats (Figure 1a,c). In the FS model rat, the collagen fiber pattern was disrupted, which confirmed capsule fibrosis. The assessment results had to be completed as soon as possible for the subsequent experiment; therefore, capsule staining was conducted. In the next histological evaluation, the capsule with the glenohumeral joint was decalcified for two months, followed by H&E staining and Masson staining, to confirm the effectiveness of Tuina.

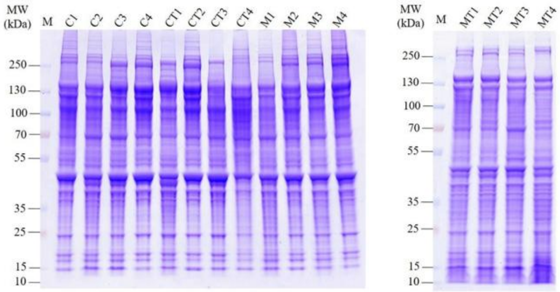

Supplementary figure 3. SDA-PAGE demonstrated that the protein bands of control samples were clear, the distribution of protein bands was normal, the protein was not degraded, the electrophoretic behavior was consistent, and high-abundance proteins were clearly visible, meeting the experimental needs.
